# Bridging the gap: the impact of parental education and child leisure activities on cognitive and socioemotional skills in preschoolers

**DOI:** 10.3389/fpsyg.2025.1568020

**Published:** 2025-08-25

**Authors:** Arina Shatskaya, Kristina Tarasova, Irina Surilova

**Affiliations:** ^1^Department of Educational Psychology and Pedagogy, Faculty of Psychology, Lomonosov Moscow State University, Moscow, Russia; ^2^Laboratory of Childhood Psychology and Digital Socialization, Federal Scientific Center of Psychological and Multidisciplinary Research, Moscow, Russia

**Keywords:** family SES, parents, media use, screen time, reading, executive functions, emotion comprehension, preschool children

## Abstract

Family socioeconomic status is broadly acknowledged to be associated with child development and wellbeing. However, the extent of this association across various dimensions of child development remains a topic of ongoing debate. This study aims to investigate the relationship between parental education and child cognitive and socioemotional skills, as well as the mediating role of children's leisure time activities, including screen time and shared book reading. The study involved 1,288 preschool children (*M* = 70.4 months, SD = 4.53) and their parents. Children's executive functions, emotion comprehension, and peer acceptance were assessed. Parents provided information regarding their educational levels; their children screen time duration and frequency of shared book reading. The conducted assessment of direct and indirect effects through path analysis revealed following findings. First, parental education is related to children's verbal working memory, cognitive flexibility, and emotional comprehension. Second, it showed no significant relation to children's peer acceptance, visual working memory, or inhibitory control. Third, shared book reading and screen time can statistically significantly explain differences in verbal working memory between children, including those associated with differences in parental education. Therefore, low reading frequency and high screen time, often observed in families with lower parental educational attainment, may serve as potential sources of disparities in children achievement and psychological wellbeing throughout development.

## 1 Introduction

Preschool age is a crucial period for the development of various key mental abilities, including cognitive, social, and emotional skills that are essential for a child's future academic and social success (Guhn et al., 2016). The level of cognitive and socioemotional skills in preschoolers may vary based on parental socioeconomic status (SES) (Letourneau et al., 2013). Social disparity is extensively documented to have a deleterious effect on the mental wellbeing of children. Those from disadvantaged backgrounds are 2–3 times more likely to experience mental health issues compared to their more advantaged peers (Kirkbride et al., 2024). There is evidence linking SES to various general preschool cognitive (e.g., Christensen et al., 2014; González et al., 2024; Incognito et al., 2022; Larson et al., 2015; Norfadillah et al., 2017), social and emotional abilities (e.g., Hosokawa and Katsura, 2018; Liu et al., 2020; Lechner et al., 2021; Wu et al., 2020). However, much less attention has been paid to examining the relations between specific components of these abilities and specific dimensions of SES (Cuartas et al., 2022; Waters et al., 2021). This complicates the development of targeted interventions for specific skills in children from specific SES backgrounds. In the current study, we focus on parental education as one of the main indicators of SES and its association with specific cognitive, social, and emotional skills discussed below.

Firstly, one of the most significant cognitive emergences during preschool age is the development of executive functions (EF). EF is a group of cognitive skills that are required for the conscious, top-down control of action, thought, and emotions (Diamond, 2013). Core EF skills include working memory—visual and verbal, inhibitory control, and cognitive flexibility (Miyake et al., 2000; Diamond, 2013; Veraksa et al., 2021a). Visual and verbal working memory facilitates the storage and manipulation of information that is not currently being perceived. Inhibitory control suppresses impulsive responses in favor of task-relevant responses, while cognitive flexibility supports the ability to switch between competing responses, rules, or perspectives. At the same time, EF can be considered more broadly and also include other skills, such as attention, planning, reasoning, fluency, and problem-solving (Diamond, 2013; Welsh et al., 1991). Children with stronger EF show greater proficiency in pursuing long-term goals, processing information, and managing social behavior in classroom settings (Diamond, 2013). EF is critical predictors of development of cognition, successful adaptation and educational achievement both in school and later in life (Vygotsky, 1983; Willoughby et al., 2012). This explains the increased interest in their study.

Research indicates that children from lower SES backgrounds perform worse than their peers on a variety of EF tasks, including working memory, flexibility, attention, and planning (Cuartas et al., 2022; Lipina et al., 2005; Hackman et al., 2015). The meta-analysis by Lawson and colleagues demonstrated a small but statistically significant correlation between SES and EF across all studies they examined [r random = 0.16, 95% CI (0.12, 0.21)] (Lawson et al., 2018). Halse et al. (2019) reported that after adjusting for all time-invariant unmeasured confounders, higher parental education predicts superior EF development. However, this study used teachers' assessments of children's EF with the BRIEF questionnaire, instead of objectively measuring the specific EF skills in the children themselves. Significantly fewer studies have investigated the relationship between specific EF skills and parental education. Ardila et al. (2005) demonstrated that based on parental education, no significant differences were observed in core EF skills. Significant associations were found for semantic and phonemic verbal fluency scores as supplementary EF measures (Ardila et al., 2005). In the work of Hackman and colleagues, parental education significantly predicted planning abilities by first grade, but not working memory (*p* > 0.05) (Hackman et al., 2015). However, Conway et al.'s (2018) study showed that the greatest differences in children's EF skills due to differences in parental education were observed for working memory. Thus, further research is needed due to the inconsistency of results and the lack of data on the relationship between specific, objectively measured EF skills and parental education.

Secondly, in addition to cognitive emergences during preschool age, social skills are also important for future academic and social success (Burt and Roisman, 2010; Moffitt et al., 2011). One of the most predictive social measures is the peer acceptance as the degree to which a child is socially accepted by his or her peers (Doll, 1996). Peer acceptance and rejection are commonly assessed through sociometric nominations, where children identify their schoolmates whom they “like most” and “like least” (McDonald and Asher, 2018). Peer acceptance is related to increased motivation for engaging in learning activities (e.g., Holmes et al., 2016), associated with improved academic performance (e.g., Ladd et al., 2017), and future psychological wellbeing (e.g., van der Wilt, 2024).

The relationship between family SES and peer acceptance has been studied to a much lesser extent than with cognitive skills. Higher parental education is associated with greater social interaction skills in children (Hoglund and Leadbeater, 2004). Kassim and Hutagalung (2020) showed that social interaction was higher in preschoolers whose parents had a higher level of education. In turn, children with poor social interaction skills become rejected more often (Ferris, 2019). Very few studies have directly examined the relationship between parental education and a child's peer acceptance. Uribe et al. (2014) found that parental education level contributes to the peer acceptance of children. As shown by Shehu (2019), the largest percentage of children accepted by their peers had at least one highly educated parent, while all children of parents with a lower level of education were categorized as rejected. However, parental education is not a strong predictor of peer acceptance in preschool (Shehu, 2019). This, along with the limited research on the connection between parental education and peer acceptance, highlights the necessity of addressing this gap.

Thirdly, the emotional skills developed during preschool age are also a significant predictor of a child's future successes (Guhn et al., 2016). Among the numerous emotional skills, understanding emotions holds particular value. Understanding emotions reflects the direct comprehension of one's own and others' emotions and the recognition and description of emotional states. It also includes cognitive aspects such as explaining the nature and causes of emotions, predicting emotions, and knowing and applying strategies for regulating them in everyday life (Pons et al., 2004). Emotional understanding is important for academic or developmental outcomes. Emotionally competent children build better relationships with teachers and peers, enhancing their engagement in learning and social activities (Guhn et al., 2016). Among all emotional competencies, emotion understanding generally shows a stronger association with overall cognitive functions (Brackett et al., 2011). Previous research has indicated that emotional understanding is related to academic performance in language and mathematics (da Silva, 2012; Rocha, 2016), positively impacts academic success (Blankson et al., 2013; Józsa and Barrett, 2018), and enhances the ability to decenter (Leerkes et al., 2008).

In research on the connection between SES and emotional skills, studies on the topic of negative socio-emotional development such as behavioral problems prevail (e.g., Japar, 2017; Kiernan and Mensah, 2009). Regarding positive emotional skills as understanding emotions, studies show that children from a lower social class had a lower level of emotion understanding (Kårstad, 2016). Cutting and Dunn (1999) demonstrated that children's understanding of emotions is linked to certain aspects of family background, particularly the education of their mothers. However, parental education made a unique contribution to false belief understanding in this study, but not to emotion understanding. Merz et al. (2015) found that expressive, receptive, and situational understanding of emotions by preschoolers was significantly correlated with parental education, which was used as a control variable in the study. Most studies regard parental education as a control variable rather than an independent factor. This underscores the need to specifically examine its relationship with emotion understanding.

Thus, there is evidence linking parental education to EF, peer acceptance, and emotional understanding. However, these relationships often remain ambiguous and fundamental aspects of this relations remain unknown (Hackman et al., 2015). This highlights the need to explore mediators that may clarify the relationships between parental education and the child EF, peer acceptance, and emotional understanding, while addressing differences associated with family SES. Unlike previous studies that focused on material resources, we propose using Vygotsky's cultural-historical theory to identify these mediators (Vygotsky and Cole, 1978). This concept considers the social environment as a source of development of higher psychological functions in humans (Veresov, 2024). Here, parent-child interaction and the organization of the children's activities can be considered as one of the main factors associated with child development (Bergman et al., 2021; González-Moreno et al., 2014; Hedegaard, 2011; Roy-Charland et al., 2020).

Children's activity can be operationalized through the concept of children's leisure activity. According to the latest monitoring data, the leisure activities of modern Russian preschoolers are primarily represented by home games and digital activities, followed by reading books, attending cultural events, and playing sports Bulletin of the Russian Longitudinal Monitoring Survey (RLMS-HSE) (2024). This study examines two common types of leisure activities among preschoolers: using electronic devices (phones, tablets, game consoles, and televisions) and shared book reading.

Using electronic devices is traditionally assessed via screen time measures. Screen time for young children (Kwon et al., 2024) and age of first interaction with electronic devices (Roy et al., 2024) are rising globally. Moreover, among low SES families, children spend considerably more time in front of screens (Kwon et al., 2024). Parental education level, as one of the indicators of SES, is significantly inversely associated with screen time among preschool-aged children (Pons et al., 2020). This is likely attributable to the fact that parents with a higher level of education, attach greater importance to limiting children's screen time (Määttä et al., 2017).

Research on the associations between screen time and child skills indicates that different types of screen time may pose risks to various child skills, including EF, emotional skills, and peer acceptance (Sticca et al., 2025; Veraksa et al., 2021a). Although the rapidly increasing number of studies on the effects of digitalization on child development, significant barriers and limitations still persist in this field. Firstly, parental compliance with recommended screen time limits for children remains low (Gago-Galvagno et al., 2024). Secondly, despite numerous studies on the association of screen time with various child competencies, most existing screen time recommendations are based on very little evidence (Sticca et al., 2025). Finally, most research on screen time and SES effects on it have focused on high-income Western countries. It emphasizes the necessity for studies addressing SES-dependent patterns across diverse sociocultural settings (Gago-Galvagno et al., 2024; Roy et al., 2024). These factors emphasize the importance of examining screen time and its mediating role between SES indicators and key cognitive and socio-emotional skills in preschool children.

Shared book reading is a highly effective early intervention for supporting children's development, as it fosters more interactive conversations than other activities (Read et al., 2022; Kameneva-Lyubavskaya and Borzova, 2024). According to Vygotsky's cultural-historical theory (1978), parents can promote children's development of social and emotional skills through shared book reading (Bergman et al., 2021; Roy-Charland et al., 2020). Children's recollections or reflections on what they have read with their parents are rich sources of conversations about emotions across cultures and contribute to the development of emotional understanding (Carmiol and Schröder, 2019; Xie et al., 2018) and self-regulation competencies (Salmon and Reese, 2016). For example, children aged 3–6 years with less frequent shared reading had a higher risk of social-emotional problems (Martin et al., 2022). For EF, introducing the practice of reading books with built-in cognitive exercises to preschoolers led to significant improvements in their working memory and cognitive flexibility, but not in inhibition (Howard et al., 2017). Studies examining the relationship between the frequency of shared reading and parental education reveal a moderately positive correlation (e.g., Pfost and Heyne, 2023), while other research indicates no differences in family reading frequency based on parental education (e.g., Curenton and Justice, 2008).

Therefore, the organization of children's leisure activities (such as facilitating shared book reading and managing screen time during digital leisure) can be linked to various aspects of a child's cognitive, social, and emotional skills.

This study has formulated two research questions. Are preschool children's key cognitive (executive functions) and socio-emotional skills (emotional understanding and peer acceptance) related to parental education (RQ1)? Can the relations between parental education and child cognitive and social-emotional skills be explained through leisure activities, particularly shared reading and screen time (RQ2)? Based on the above literature review, we hypothesized affirmative answers to both research questions.

## 2 Materials and methods

### 2.1 Participants and procedure

A total of 1,288 preschool children (Age: *M* = 70.4 months, SD = 4.53; 49.5% girls) and their parents (Age: *M* = 35 years, SD = 7) participated in the study on a voluntary basis. Participants were recruited from penultimate preschool groups before school of Russian kindergartens in Moscow (74.4%), Kazan (12.6%), and Sochi (13%). All participants were recruited from urban areas and spoke Russian as their first language. Parents provided written consent for their children's involvement in the research. All children were assessed as not having developmental delays or disabilities. The assessment tasks were administered individually within a quiet space in the kindergarten during the two morning sessions. Children had the option to decline participation if they felt uncomfortable. After completing the assessment, each child received a small, enjoyable sticker as a reward.

At the same time, the parents completed a parental survey that included questions about the parents' level of education and a block of questions about the child's leisure time activities: the child's screen time and the frequency with which books were read to the child.

### 2.2 Measures

#### 2.2.1 Cognitive skills

Children's executive function (EF), encompassing visual and verbal working memory, inhibitory control, and cognitive flexibility, was assessed using the NEPSY-II neuropsychological test battery (Korkman et al., 2007). Visual working memory was measured through the “Memory for Design” subtest, requiring children to recall the correct arrangement of image components (max score: 150). Verbal working memory was evaluated using “Sentence Repetition” where participants listened to and repeated a series of increasingly complex sentences (max score: 34).

Inhibitory control was assessed with the “Inhibition” subtest, which involved two tasks: “Naming,” where children quickly identified all figures, and “Inhibition,” where they named figures in reverse (e.g., stating “square” for “circle”) (max score: 20). Cognitive flexibility was measured using the “DCCS” tool, consisting of three tasks: sorting cards by color, sorting by shape, and sorting by a specific rule based on card framing (max score: 24) (Zelazo, 2006). These EF assessment tools were adapted for the Russian population, proving their reliability (Veraksa et al., 2020).

#### 2.2.2 Socioemotional skills

The emotional comprehension of the child was assessed using the Test of Emotion Comprehension (TEC) (Pons and Harris, 2000), which was adapted for the Russian context, proving its reliability (Veraksa et al., 2021b). TEC evaluates three aspects of emotion comprehension: external components (recognizing emotions, understanding external causes, and desires), mental components (grasping hidden emotions and the role of beliefs), and meta-components (understanding mixed feelings and moral influence on emotional regulation). Scores from these scales are combined for a comprehensive emotion understanding score ranging from 0 to 9.

The child's peer acceptance was measured using Kolominsky's sociometric status tests (Kolominsky, 1984). Children were asked to answer questions regarding their relationships with peers from their kindergarten group by naming three classmates: “Who do you like to play with? Choose three children from your group, please.” The sociometric status was determined based on the number of play partner selections received. This procedure is a reliable measure of peer status (Wasik, 1987).

#### 2.2.3 Children's leisure activities

Child screen time was evaluated through four questions in the caregiver survey. Parents reported the duration of child passive screen time (watching cartoons, films, and videos) on weekdays and weekends, as well as active screen time (using devices for non-viewing activities) during the same periods. The parent specified the number of hours and minutes as a number, for example the total time is: 1 (h) and 15 (min). Daily screen time was calculated as the sum of passive and active screen time on weekdays and weekends divided by the number of days in a week.

Book reading frequency was assessed by asking caregivers how often they read with their child. Parents selected one of the following response options: once or several times a day, almost every day, 2–3 times a week, several times a month, or not at all.

#### 2.2.4 Parental education

Parental education was measured by asking caregivers about their highest level of education attained, with options including: primary school, basic general school, secondary school, vocational secondary education, bachelor's degree, specialist degree, master's degree, and PhD.

### 2.3 Data analytical strategy

Preliminary analyses included simple correlation analyses corrected for age and sex at birth. Additional analyses were then conducted to assess the significance of differences in shared reading frequency and screen time between groups of children with different levels of parental education. Specifically, Chi-squared tests were used to compare shared reading frequencies. Kruskal-Wallis test as a non-parametric method for comparing medians across multiple independent groups (Cleophas and Zwinderman, 2016) was employed to evaluate differences in daily screen time.

In the main analysis, Path analyses with bootstrap sampling (1,000 samples) were estimated to assess the direct effects of parental education on children's cognitive and socioemotional skills and the indirect effects mediated by daily screen time and shared book reading. To answer the research questions, path analysis was chosen as the main method. It is more advantageous than linear regression analysis because it offers an understanding of the relationships and the relative significance of each factor, while also exploring the direct and indirect connections among the variables. The mediator variable transmits part of the effect of the causally prior variable to a third variable influenced by the mediator, as defined in traditional mediation analysis by Kline (2016). It assumes that the variables are ordered temporally (a condition necessary to establish causal relationships). This process serves to elucidate the nature of the relationship between the independent and dependent variables (MacKinnon, 2008). However, since the current data are exploratory and lack the temporal ordering required to establish causality and properly test mediation, the resulting estimates may be biased (Maxwell and Cole, 2007). All analyses were conducted in R (version 4.4.0) and Jamovi (version 2.3.16).

## 3 Results

### 3.1 Descriptive statistics and preliminary analysis

Descriptive statistics for main continuous study variables (EF skills, sociometric status, emotion comprehension, screen time) are summarized in Table 1. The frequency distribution by parental education shows that 5% of parents have completed basic general school education, 17.3% have completed vocational secondary education, 12.4% have completed a bachelor's degree, 62.7% have completed a master's degree and 2.6% have a PhD. The frequency distribution of reading to children indicated that 34.7% of parents read books to their children once or several times a day; 20.8% read almost every day; 25.6% read 2–3 times a week; 17.7% read several times a month; 1.2% do not read at all.

**Table 1:** Descriptive statistics for children EF skills, emotion comprehension, sociometric status, and screen time dimensions.

**Study variables**	**Mean**	**Median**	**SD**	**Minimum**	**Maximum**	**Skewness**	**SE Skewness**	**Kurtosis**	**SE Kurtosis**
Visual working memory	88.41	88.00	21.812	17	130	−0.183	0.045	−1.031	0.0898
Verbal working memory	20.13	20.00	4.540	0	34	−0.142	0.044	0.586	0.0886
Inhibition	11.37	11.00	3.212	1	19	−0.140	0.046	−0.237	0.0911
Cognitive flexibility	21.29	22.00	2.513	3	24	−1.150	0.044	1.824	0.0877
Sociometric status	1.85	2.00	0.507	1	3	−0.235	0.048	0.441	0.0968
Emotion comprehension	5.75	6.00	1.417	0	12	−0.223	0.046	0.021	0.0913
Passive weekdays screen time	90.74	70	70.069	0	1,200	3.821	0.066	46.96	0.1323
Passive weekends screen time	154.23	120.00	103.758	0	1,215	2.733	0.066	18.31	0.1322
Active weekdays screen time	56.76	30.00	75.401	0	1,200	4.714	0.067	50.57	0.1344
Active weekends screen time	87.86	60	91.846	0	600	1.645	0.067	3.067	0.1343
Daily screen time	192.04	157.50	135.755	8.00	1,200	1.779	0.066	5.37	0.1321

SD, standard deviation; SE, standard error.

Table 2 displays Pearson and Spearman correlations among study variables (controlling for age and sex). Correlations between the same variables without adjustment for sex and age are shown in Supplementary Table 2 (since the path models do not include these covariates). Parental education was significantly and positively correlated with verbal working memory, cognitive flexibility, and emotion comprehension, as well as significantly and negatively correlated with screen time. Reading frequency showed similar positive correlations with verbal working memory and negative correlations with screen time dimensions. In addition, the frequency of reading to the child is associated with the level of parents' education (*r* = *0.18; p*<*0.001*).

**Table 2:** Age-sex-corrected correlations for children EF skills, emotion comprehension, sociometric status, screen time dimensions, frequency of shared book reading, and level of parental education.

**Study variables**	**Values**	**1**	**2**	**3**	**4**	**5**	**6**	**7**	**8**	**9**
Parental education (1)	Pearson's r	—								
	*p-value*	—								
	Spearman's rho	—								
	*p-value*	—								
	*N*	—								
Shared book reading (2)	Pearson's r	**0.183**	—							
	*p-value*	**< 0.001**	—							
	Spearman's rho	**0.196**	—							
	*p-value*	**< 0.001**	—							
	*N*	**1,287**	—							
Daily screen time (3)	Pearson's r	**−0.267**	**−0.225**							
	*p-value*	**< 0.001**	**< 0.001**	—						
	Spearman's rho	**−0.271**	**−0.211**	—						
	*p-value*	**< 0.001**	**< 0.001**	—						
	*N*	**1,279**	**1,356**	—						
Visual working memory (4)	Pearson's r	0.044	0	−0.014	—					
	*p-value*	0.125	0.992	0.632	—					
	Spearman's rho	0.034	−0.002	−0.001	—					
	*p-value*	0.237	0.934	0.97	—					
	*N*	1,212	1,217	1,218	—					
Verbal working memory (5)	Pearson's r	**0.213**	**0.112**	**−0.207**	0.298	—				
	*p-value*	**< 0.001**	**< 0.001**	**< 0.001**	< 0.001	—				
	Spearman's rho	**0.182**	**0.103**	**−0.22**	0.295	—				
	*p-value*	**< 0.001**	**< 0.001**	**< 0.001**	< 0.001	—				
	*N*	**1,245**	**1,250**	**1,250**	2,928	—				
Inhibition (6)	Pearson's r	0.035	0.037	−0.039	0.279	0.219	—			
	*p-value*	0.23	0.203	0.175	< 0.001	< 0.001	—			
	Spearman's rho	0.028	0.028	−0.006	0.274	0.203	—			
	*p-value*	0.34	0.323	0.831	< 0.001	< 0.001	—			
	*N*	1,201	1,207	1,209	2,706	2,733	—			
Cognitive flexibility (7)	Pearson's r	**0.066**	0.027	0.013	0.306	0.289	0.242	—		
	*p-value*	**0.019**	0.343	0.646	< 0.001	< 0.001	< 0.001	—		
	Spearman's rho	**0.061**	0.021	0.006	0.315	0.31	0.241	—		
	*p-value*	**0.03**	0.457	0.827	< .001	< .001	< .001	—		
	N	**1,272**	1,282	1,284	2,945	2,975	2,828	—		
Sociometric status (8)	Pearson's r	0.05	−0.025	0.007	0.102	0.101	0.085	0.085	—	
	*p-value*	0.087	0.402	0.819	< 0.001	< 0.001	< 0.001	< 0.001	—	
	Spearman's rho	0.036	−0.025	−0.006	0.098	0.102	0.08	0.094	—	
	*p-value*	0.225	0.402	0.835	< 0.001	< 0.001	< 0.001	< 0.001	—	
	*N*	1,149	1,157	1,160	2,289	2,326	2,272	2,415	—	
Emotion comprehension (9)	Pearson's r	**0.078**	0.006	**−0.073**	0.193	0.18	0.118	0.184	0.029	—
	*p-value*	**0.007**	0.843	**0.012**	< 0.001	< 0.001	< 0.001	< 0.001	0.174	—
	Spearman's rho	0.056	−0.001	**−0.064**	0.186	0.178	0.103	0.199	0.028	—
	*p-value*	0.056	0.97	**0.028**	< 0.001	< 0.001	< 0.001	< 0.001	0.185	—
	*N*	1,184	1,187	**1,188**	2,769	2,812	2,740	2,819	2,219	—

Bold indicates significant values (*p* < 0.05).

### 3.2 Parental education and children's leisure activities

Based on the results of the age-sex-corrected correlation analysis presented in Table 2, a higher parental education level was found to be associated with greater book reading to children and less daily screen time for children. Further Chi-square analyses confirmed significant disparities in book reading frequency between parents with different levels of education [χ^2^(56) = 112, *p* < 0.001]. Kruskal-Wallis analyses also confirmed significant differences in daily screen time for children: parents with a higher level of education had children with significantly lower daily screen time [χ^2^(7) = 120, *p* < 0.001, ε^2^ = 0.09].

### 3.3 Children's leisure activities and their cognitive and socioemotional skills

#### 3.3.1 Digital devices screen time

Correlation analysis showed that daily screen time was significantly negatively associated with verbal working memory and emotion comprehension (Table 2). More screen time is linked to poorer verbal working memory and emotion comprehension in children.

#### 3.3.2 Shared book reading

Correlation analysis demonstrated that book reading frequency was exclusively associated with verbal working memory (see Table 2). Greater frequency of shared reading was found to be associated with higher verbal working memory.

### 3.4 Direct and indirect effect of parental education on child cognitive and socioemotional skills through shared book reading and daily screen time mediation

**Figure 1:**
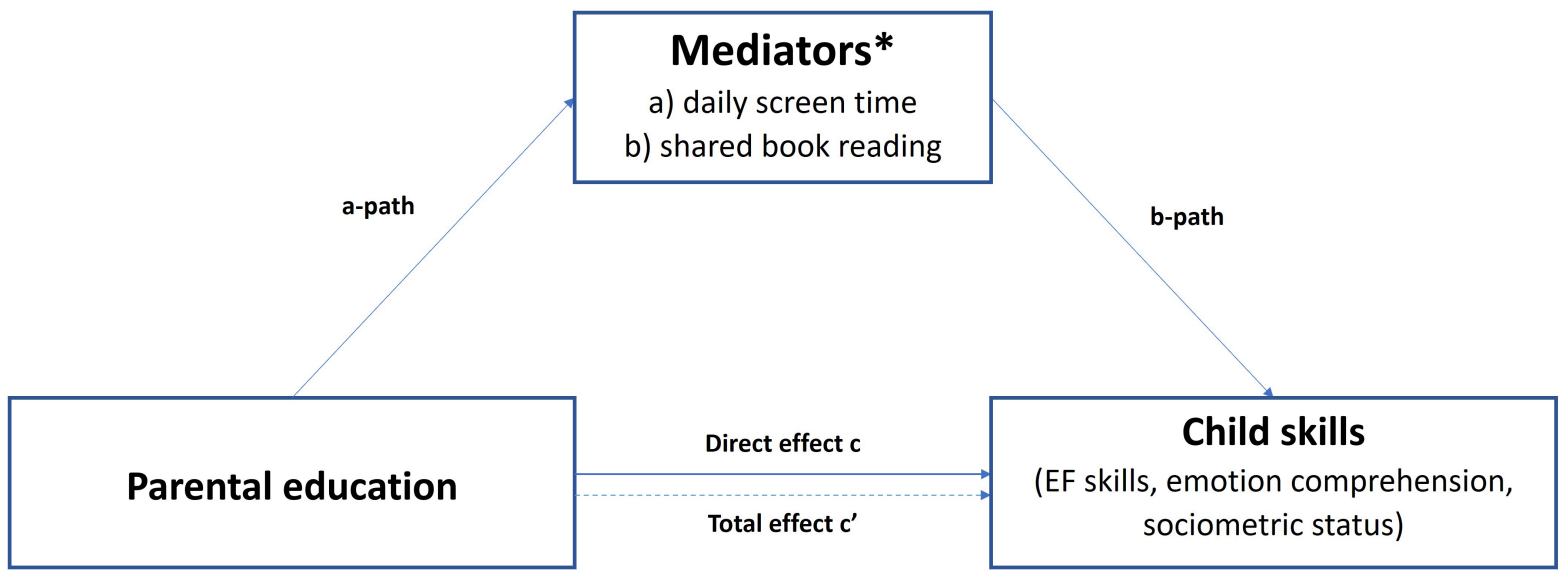
Path analysis models for measuring direct and indirect effect of parental education on child cognitive and socioemotional skills. *Models of direct effects were developed to examine the relations between parental education and EF skills (visual working memory, verbal working memory, inhibitory control, cognitive flexibility), sociometric status, and emotions comprehension. Additionally, 12 models of indirect effects were constructed to connect these variables: six models utilizing (a) daily screen time as a mediator and six models utilizing (b) shared book reading as a mediator.

First, six path analysis models were specified to assess both the direct effects of parental education on children's EF skills, sociometric status, and emotion comprehension, as well as the indirect effects operating through daily screen time (Figure 1). The analysis of direct effects showed that higher parental education was significantly associated with higher verbal working memory (*Z* = *5.41, p* < *0.001*), cognitive flexibility (*Z* = *1.88, p* = *0.049*), and emotion comprehension (*Z* = *2.57, p* = *0.01*) (Table 3). The analysis of indirect effects (through daily screen time) showed that daily screen time significantly mediated the relationship between parental education and verbal working memory (*Z* = *3.95, p* = < *0.001*, 21.3% mediation) (Table 3).

**Table 3:** Direct and indirect [through (a) daily screen time as mediator and (b) shared book reading as mediator] association between parental education and child skills (EF skills, emotion comprehension, sociometric status).

**Child skills**	**Direct effect (c)**				**Indirect effect (a**×**b)**			
	**Estimate (95% low, high C.I.)**	**SE**	***Z*** **(p)**	**% mediation**	**Estimate (95% low, high C.I.)**	**SE**	***Z*** **(p)**	**% mediation**
**Daily screen time as mediator**								
Visual working memory	0.89 (−0.08, 1.88)	0.49	1.81 (0.07)	97.52	−0.02 (−0.27, 0.29)	0.13	−0.17 (0.863)	2.48
Verbal working memory	0.59 (0.38, 0.81)	0.11	**5.41 (< 0.001)**	78.7	0.16 (0.08, 0,25)	0.04	**3.95 (< 0.001)**	21.3
Inhibition	0.07 (−0.08, 0.22)	0.07	1.0 (0.317)	75.9	0.02 (−0.01, 0.06)	0.01	1.32 (0.186)	24.1
Cognitive flexibility	0.10 (0.01, 0.21)	0.05	**1.88 (0.049)**	95.4	−0.01 (−0.03, 0.03)	0.02	−0.33 (0.744)	4.42
Sociometric status	0.02 (−0.01, 0.05)	0.01	1.69 (0.092)	92.6	−0.01 (−0.01, 0.1)	0.01	−0.55 (0.585)	7.37
Emotion comprehension	0.07 (0.01, 0.13)	0.03	**2.57 (0.01)**	83.1	‘0.02 (0.0, 0.03)	0.01	1.98 (0.057)	16.9
**Shared book reading as mediator**								
Visual working memory	0.82 (−0.18, 1.80)	0.50	1.64 (0.099)	99.9	0.00 (−0.18, 0.17)	0.08	−0.01 (0.995)	0.1
Verbal working memory	0.68 (0.48, 0.89)	0.1	**6.49 (< 0.001)**	92.14	0.06 (0.02, 0.11)	0.02	**2.77 (0.006)**	7.86
Inhibition	0.08 (−0.05, 0.22)	0.07	1.18 (0.248)	83.6	0.01 (−0.007, 0.03)	0.01	1.38 (0.167)	16.4
Cognitive flexibility	0.95 (−0.005, 0.19)	0.05	**1.79 (0.044)**	92.53	0.007 (−0.01, 0.02)	0.008	0.87 (0.383)	7.47
Sociometric status	0.02 (−0.001, 0.04)	0.01	1.87 (0.061)	88.0	−0.002 (−0.01, 0.01)	0.02	−1.22 (0.223)	11.1
Emotion comprehension	0.08 (0.03, 0.14)	0.02	**3.1 (0.002)**	99.6	0.001 (−0.01, 0.01)	0.005	0.06 (0.948)	0.397

CI, confidential interval; SE, standard error; c, direct effect (Figure 1), a × b, indirect effect (Figure 1). Bold indicates significant values (*p* < 0.05).

Second, six models of the relationship between parental education and children's skills through shared book reading were conducted (see Figure 1). The analysis of direct effects showed similar results: higher parental education was significantly associated with higher verbal working memory (*Z* = *6.49, p* < *0.001*), cognitive flexibility (*Z* = *1.79, p* = *0.044*), and emotion comprehension (*Z* = *3.1, p* = *0.002*) (Table 3). The analysis of indirect effects (through shared book reading) showed that shared book reading also significantly mediated the relationship of parental education with verbal working memory only (*Z* = *2.77, p* = *0.006*, 7.86% of mediation) (Table 3). For all Direct Effects models and Partial Mediation models, the CFI and TLI indices were 1, RMSEA and SRMR were 0. Fit indices for each mediation model test (RMSEA, CFI, TLI, and SRMR) can be seen in Supplementary Table 1. Thus, child screen time and frequency of shared book reading can be considered as a potential factor explaining the relationship between parental education and verbal working memory.

## 4 Discussion

The extent to which a child's environment supports or limits their developmental potential is closely linked to various indicators of socioeconomic status, including parental education. Despite extensive research in SES field, not all studies identify significant associations between SES and various child skills. Furthermore, a meta-analysis by Letourneau et al. (2013) shows that SES association has effect sizes ranging from very small to small. Given this, this study examines the association between parental education, one of the key SES indicators, and cognitive and socioemotional skills in preschoolers. Another key aim of this study was to examine if children's leisure activities, such as screen time and shared book reading, mediate the relationship between parental education and children's cognitive and socioemotional skills.

By estimating direct effects in path analysis, parental education was shown to be directly related to several child skills, including verbal working memory, cognitive flexibility, and emotion comprehension. In contrast, the peer acceptance, visual working memory, and inhibitory control showed no significant relationship with parental education. The link between child skills and parental education may be because parents with higher education invest more in their children's development than those with lower education or economic resources (Bergen et al., 2017). Parents with higher education are better able to involve their children in educational activities that build a strong foundation for future success (Davis-Kean et al., 2021).

The lack of significant relations of parental education level with visual working memory and inhibitory control, in contrast to the small but significant association with verbal working memory and cognitive flexibility, may be explained. Parents with higher education levels create a more intellectually stimulating environment for their children, especially in terms of language, by using richer vocabulary and reading to them more often (Hoff and Laursen, 2019; Hoff-Ginsberg, 1991). Parents with higher education levels may create environments that boost language development in their children. This enhanced language development is linked to improved verbal working memory and cognitive flexibility, though not to visual working memory or inhibition. Several studies support this supposed explanation. For example, Filipe et al. (2023) found that, after accounting for age, gender, and nonverbal intelligence, verbal working memory, and cognitive flexibility explained 16% and 19% of the differences in preschoolers' language skills, respectively. Inhibition skills did not add to the explained variance in language outcomes (Filipe et al., 2023; Oshchepkova and Shatskaya, 2023). At the same time, meta-analysis data by Pickering et al. (2023) showed that language development in children aged 2–12 years has weak links with visuospatial memory.

Regarding socioemotional skills, the analysis found a significant association between parental education and emotional understanding but not peer acceptance. On the one hand, significant association of parental education with peer acceptance is expected. Research indicates that poorly educated parents tend to be more isolated, less involved in the school system, and provide fewer opportunities for continuous learning for their children outside the school environment (McLoyd, 1998). In such families, children may have limited opportunities to develop interpersonal skills, which can negatively affect peer acceptance and increase emotional-behavioral problems (Dodge et al., 1994). In this context, the lack of an association between parental education and peer acceptance in the current study might be due to insufficient diagnostic procedures for assessing peer acceptance. Future studies could expand assessment instruments by, for example, asking participants about the motives for choosing their peers. On the other hand, Bronfenbrenner's bioecological theory suggests that parental education serves as only one contextual external factor in children's development (Bronfenbrenner and Morris, 2006). Thus, the lack of connection between peer acceptance and parental education can also possibly be explained by the influence of some stronger factors that neutralize the connection with SES indicators (e.g., children's individual characteristics or the nature of parent-child interactions).

Thus, the current analysis confirmed the association of parental education with some skills of children in the Russian sample. The long-term nature of this association is also confirmed in longitudinal studies. For example, early family income-to-needs and maternal education predicted EF by first grade (Hackman et al., 2015). At the same time, the relationship between parental education and EF emerges in early childhood and remains stable later in life (Hackman et al., 2014).

Although research over several decades has clearly documented differences in children's skills due to SES, there is a noticeable lack of policies or interventions that reliably reduce these disparities (Davis-Kean et al., 2021). This highlights the importance of analyzing factors that mediate the relationships between SES indicators and children's skills. The second research question was directed at mediators of the relationship between parental education and child cognitive and socioemotional skills. Parents were asked a few brief questions about the duration of their children's screen time and the frequency of shared book reading. The evaluation of indirect effects in path analysis indicated that the connection between parental education and verbal working memory is partially explained by child screen time and shared book reading frequency (21% and 8%, respectively). Children of more educated parents experience higher frequency of shared reading and lower screen time. This is positively linked to children's verbal working memory. It is important to acknowledge one limitation of the mediation models conducted. The mediation models were analyzed without including covariates such as sex and age, which might have affected the results to some extent. However, comparing simple correlations between parental education and children's EF skills, emotional comprehension, sociometric status, and screen time (see Supplementary Table 2) with age- and sex-corrected correlations (see Table 2) shows only slight differences. This suggests that age and sex have a small impact on these relationships. Therefore, mediation models can be used without including these covariates. Nevertheless, it is advisable for future studies to include sex and age in mediation models to strengthen the conclusions.

We assume that the presence of the most significant mediation for verbal working memory is explained by the following. Verbal working memory is associated with the language environment (Schwering and MacDonald, 2020). When listening to reading, the child's language input increases Noble et al., (2018). On the contrary, passive viewing of video content, as the most common type of digital activity, reduces children's speech and communicative activity (Massaroni et al., 2024).

However, using questions only about screen time duration and frequency of shared reading has limitations. This method does not capture the qualitative aspects of children's leisure activities (the content of the reading sessions, who initiates them, or details about screen time like the types of media accessed and children's preferences). Previous studies have highlighted the importance of these qualitative features. For example, the average level of EF skills varied depending on the purpose behind the child's use of digital devices (Shatskaya et al., 2023) or the type of reading, such as traditional reading, dialogic reading, or reading that involves embedded cognitive activities (Howard et al., 2017). Controlling for content (educational or entertainment), the negative effects of screen time on children's skills can be reduced (Linebarger et al., 2014; Yang et al., 2017). The content of books for shared reading also matters. (Noble et al. 2018) showed that books with richer grammar lead to more complex adult speech to children, which benefits their development. A deeper understanding of the content and context of media use and shared reading could help identify different connections to developmental outcomes.

Although this study is correlational, its results may guide future longitudinal research. Such studies could examine how adjusting reading frequency and screen time might reduce the negative impact of low parental education on children's cognitive and socio-emotional skills.

### 4.1 Strengths and limitations

The most valuable advantage of the current study is its focus on non-Western countries, while most studies of SES effects have been conducted on samples from Western countries. At the same time, the study has several limitations. Firstly, the design is cross-sectional, limiting our ability to establish causal relationships and allowing only for descriptions of associations among the factors studied. Additionally, this cross-sectional nature reinforces the exploratory character of the mediation analysis, as it does not provide the temporal ordering necessary to definitively test for mediation effects. Secondly, we utilized a short questionnaire for parents that quantitatively assess the frequency of shared book reading and the duration of screen time. This approach lacks depth, as it does not explore qualitative aspects of children's leisure activities, such as the specific content being read, who initiates the reading, or details regarding screen time, including the types of media consumed and children's preferences. A more nuanced understanding of the content and context of media use and shared reading could reveal differential relationships with developmental outcomes. Third, social desirability of parents' responses to the questionnaire may also bias the results. Future research could address these limitations by using in-depth interviews with parents to gain a more comprehensive understanding of these practices.

## 5 Conclusions

Parental education, as a crucial indicator of family socioeconomic status, is associated with verbal working memory, cognitive flexibility, and emotion comprehension. However, was found to lack a statistically significant association with peer acceptance, as well as visual working memory and inhibitory control. Meanwhile, shared book reading and screen time control seem to be able to statistically significantly explain differences in verbal working memory between children, including those associated with differences in parental education. It is highly recommended to provide health education for parents on effectively organizing children's leisure time, which includes setting reasonable limits on screen time for preschoolers and increasing the frequency of shared book reading.

## Data Availability

The raw data supporting the conclusions of this article will be made available by the authors, without undue reservation.

## References

[B1] Ardila A. Rosselli M. Matute E. Guajardo S. (2005). The influence of the parents' educational level on the development of executive functions. Dev. Neuropsychol. 28, 539–560. 10.1207/s15326942dn2801_5 15992255

[B2] Bergen E. Zuijen T. Bishop D. Jong P. F. (2017). Why are home literacy environment and children's reading skills associated? What parental skills reveal. Read. Res. Q. 52, 147–160. 10.1002/rrq.160

[B3] Bergman D. Aram D. Khalaily-Shahadi M. Dwairy M. (2021). Promoting preschoolers' mental-emotional conceptualization and social understanding: a shared book-reading study. Early Educ. Dev. 32, 501–515. 10.1080/10409289.2020.1772662

[B4] Blankson A. N. O'Brien M. Leerkes E. M Marcovitch S. Calkins S. D. Weaver J. M. (2013). Developmental dynamics of emotion and cognition processes in preschoolers. Child Dev. 84, 346–360. 10.1111/j.1467-8624.2012.01841.x 22925076 PMC3511641

[B5] Brackett M. A. Rivers S. E. Salovey P. (2011). Emotional intelligence: implications for personal, social, academic, and workplace success. Soc. Pers. Psychol. Compass, 5, 88–103. 10.1111/j.1751-9004.2010.00334.x40101567

[B6] Bronfenbrenner U. Morris P. A. (2006). “The bioecological model of human development,” in Handbook of Child Psychology: Theoretical Model of Human Development, eds. W. Damon (Series Ed.) and Lerner R. M. (New York, NY: John Wiley), 793–828.

[B7] Bulletin of the Russian Longitudinal Monitoring Survey (RLMS-HSE) (2024). Vol. 14: Collection of Scientific Articles, ed. Kozyrev P. M.. Moscow: National Research University Higher School of Economics.

[B8] Burt K. B. Roisman G. I. (2010). Competence and psychopathology: cascade effects in the NICHD study of early child care and youth dvelopment. Dev. Psychopathol. 22, 557–567. 10.1017/S0954579410000271 20576178 PMC13152396

[B9] Carmiol A. M. Schröder L. (2019). Emotion talk during mother–child reminiscing and book sharing and children's socioemotional competence: evidence from Costa Rica and Germany. Cult. Brain 7, 126–147. 10.1007/s40167-019-00078-x

[B10] Christensen D. L. Schieve L. A. Devine O. Drews-Botsch C. (2014). Socioeconomic status, child enrichment factors, and cognitive performance among preschool-age children: results from the follow-up of growth and development experiences study. Res. Dev. Disabil. 35, 1789–1801. 10.1016/j.ridd.2014.02.003 24679548 PMC4997613

[B11] Cleophas T. J. Zwinderman A. H. (2016). “Non-parametric tests for three or more samples (Friedman and Kruskal-Wallis),” in Clinical Data Analysis on a Pocket Calculator: Understanding the Scientific Methods of Statistical Reasoning and Hypothesis Testing (Cham: Springer International Publishing), 193–197. 10.1007/978-3-319-27104-0_34

[B12] Conway A. Waldfogel J. Wang Y. (2018). Parent education and income gradients in children's executive functions at kindergarten entry. Child. Youth Serv. Rev. 91, 329–337. 10.1016/j.childyouth.2018.06.009

[B13] Cuartas J. Hanno E. Lesaux N. K. Jones S. M. (2022). Executive function, self-regulation skills, behaviors, and socioeconomic status in early childhood. PLoS ONE 17:e0277013. 10.1371/journal.pone.0277013 36322600 PMC9629624

[B14] Curenton S. M. Justice L. M. (2008). Children's preliteracy skills: influence of mothers' education and beliefs about shared-reading interactions. Early Educ. Dev. 19, 261–283. 10.1080/10409280801963939

[B15] Cutting A. L. Dunn J. (1999). Theory of mind, emotion understanding, language, and family background: individual differences and interrelations. Child Dev. 70, 853–865. 10.1111/1467-8624.00061 10446724

[B16] da Silva E. D. B. P. (2012). A relação entre inteligência emocional e o rendimento escolar em crianças do 1° Ciclo do Ensino Básico da RAM (Master's thesis). Universidade da Madeira, Portugal.

[B17] Davis-Kean P. E. Tighe L. A. Waters N. E. (2021). The role of parent educational attainment in parenting and children's development. Curr. Dir. Psychol. Sci. 30, 186–192. 10.1177/0963721421993116

[B18] Diamond A. (2013). Executive functions. Ann. Rev. Psychol. 64, 135–168. 10.1146/annurev-psych-113011-143750 23020641 PMC4084861

[B19] Dodge K. A. Pettit G. S. Bates J. E. (1994). Socialization mediators of the relation between socioeconomic status and child conduct problems. Child Dev. 65, 649–665. 10.2307/1131407 8013245

[B20] Doll B. (1996). Children without friends: implications for practice and policy. Sch. Pyschol. Rev. 25, 165–183. 10.1080/02796015.1996.12085809

[B21] Ferris E. (2019). Lessons of policing and exclusion. J. Cult. Values Educ. 2, 25–43. 10.46303/jcve.03.02.2

[B22] Filipe M. G. Veloso A. S. Frota S. (2023). Executive functions and language skills in preschool children: the unique contribution of verbal working memory and cognitive flexibility. Brain Sci. 13:470. 10.3390/brainsci13030470 36979280 PMC10046801

[B23] Gago-Galvagno L. G. Castillo M. P. Fernandez M. A. Tabullo A. J. Miller S. E. Elgier A. M.. (2024). Dyadic interactions, communication and regulation skills: associations with screen use in toddlers from Buenos Aires. Psychol. Russ. 17, 39–59. 10.11621/pir.2024.0403 40823329 PMC12352358

[B24] González L. Popovic M. Rebagliato M. Estarlich M. Moirano G. Barreto-Zarza F.. (2024). Socioeconomic position, family context, and child cognitive development. Eur. J. Pediatr. 183, 2571–2585. 10.1007/s00431-024-05482-x 38483609 PMC11098862

[B25] González-Moreno C. X. Solovieva Y. Quintanar-Rojas L. (2014). Educational policies and activities for preschool children: reflections from the cultural-historical approach and activity theory. Rev. Facul. Med. 62, 647–658. 10.15446/revfacmed.v62n4.43468

[B26] Guhn M. Gadermann A. M. Almas A. Schonert-Reichl K. A. Hertzman C. (2016). Associations of teacher-rated social, emotional, and cognitive development in kindergarten to self-reported wellbeing, peer relations, and academic test scores in middle childhood. Early Child. Res. Q., 35, 76–84. 10.1016/j.ecresq.2015.12.027

[B27] Hackman D. A. Betancourt L. M. Gallop R. Romer D. Brodsky N. L. Hurt H.. (2014). Mapping the trajectory of socioeconomic disparity in working memory: parental and neighborhood factors. Child Dev. 85, 1433–1445. 10.1111/cdev.12242 24779417 PMC4107185

[B28] Hackman D. A. Gallop R. Evans G. W. Farah M. J. (2015). Socioeconomic status and executive function: developmental trajectories and mediation. Dev. Sci. 18, 686–702. 10.1111/desc.12246 25659838

[B29] Halse M. Steinsbekk S. Hammar Å. Belsky J. Wichstrøm L. (2019). Parental predictors of children's executive functioning from ages 6 to 10. Br. J. Dev. Psychol. 37, 410–426. 10.1111/bjdp.12282 30816580

[B30] Hedegaard M. (2011). “A cultural-historical approach to children's development of multiple cultural identities,” in Children, Development and Education. International Perspectives on Early Childhood Education and Development, eds. Kontopodis M. Wulf C. Fichtner B., Vol. 3 (Dordrecht: Springer), 117–135.

[B31] Hoff E. Laursen B. (2019). “Socioeconomic status and parenting,” in Handbook of Parenting: Biology and Ecology of Parenting, ed. Bornstein M. H. (London: Routledge), 421–447.

[B32] Hoff-Ginsberg E. (1991). Mother-child conversation in different social classes and communicative settings. Child Dev. 62, 782–796. 10.2307/1131177 1935343

[B33] Hoglund W. L. Leadbeater B. J. (2004). The effects of family, school, and classroom ecologies on changes in children's social competence and emotional and behavioral problems in first grade. Dev. Psychol. 40:533. 10.1037/0012-1649.40.4.533 15238041

[B34] Holmes C. J. Kim-Spoon J. Deater-Deckard K. (2016). Linking executive function and peer problems from early childhood through middle adolescence. J. Abnorm. Child Psychol. 44, 31–42. 10.1007/s10802-015-0044-5 26096194 PMC4689661

[B35] Hosokawa R. Katsura T. (2018). Socioeconomic status, emotional/behavioral difficulties, and social competence among preschool children in Japan. J. Child Fam. Stud. 27, 4001–4014. 10.1007/s10826-018-1231-0

[B36] Howard S. J. Powell T. Vasseleu E. Johnstone S. Melhuish E. (2017). Enhancing preschoolers' executive functions through embedding cognitive activities in shared book reading. Educ. Psychol. Rev. 29, 153–174. 10.1007/s10648-016-9364-4

[B37] Incognito O. Tarchi C. Pinto G. (2022). The association between school-level SES and emergent literacy in Italy (La relación entre el nivel socioeconómico a nivel de centro escolar y la alfabetización emergente en Italia). Cult. Educ. 34, 102–139. 10.1080/11356405.2021.2006909

[B38] Japar M. (2017). Parents' education, personality, and their children's disruptive behaviour. Int. J. Instruct. 10, 227–240. 10.12973/iji.2017.10315a

[B39] Józsa K. Barrett K. C. (2018). Affective and social mastery motivation in preschool as predictors of early school success: a longitudinal study. Early Childhood Res. Q. 45, 81–92. 10.1016/j.ecresq.2018.05.007

[B40] Kårstad S. B. (2016). Young children's emotion understanding: The impact of parent and child factors, socioeconomic status, and culture (Doctoral thesis). Norwegian University of Science and Technology, Trondheim, Norway. Available online at: https://ntnuopen.ntnu.no/ntnu-xmlui/handle/11250/2409891

[B41] Kameneva-Lyubavskaya E. N. Borzova T. V. (2024). Development of metacognitive skills in teaching ways of understanding the text. Russ. Psychol. J. 21, 211–228. (in Russian).

[B42] Kassim J. Hutagalung F. D. (2020). Socioeconomic status (Ses) differences in preschoolers'social skills. J. Nusantara Stud. 5, 303–328. 10.24200/jonus.vol5iss2pp303-328

[B43] Kiernan K. E. Mensah F. K. (2009). Poverty, maternal depression, family status and children's cognitive and behavioural development in early childhood: a longitudinal study. J. Soc. Policy 38, 569–588. 10.1017/S004727940900325040682716

[B44] Kirkbride J. B. Anglin D. M. Colman I. Dykxhoorn J. Jones P. B. Patalay P.. (2024). The social determinants of mental health and disorder: evidence, prevention, and recommendations. World Psychiatry 23:58. 10.1002/wps.21160 38214615 PMC10786006

[B45] Kline R. B. (2016). Principles and Practice of Structural Equation Modeling, 4th Edn. New York: Guilford Press.

[B46] Kolominsky Y. L. (1984). Psychology of the Children's Collective (in Russian). Minsk: Narodnaya Asveta.

[B47] Korkman M. Kirk U. Kemp S. L. (2007). NEPSY II: Administrative Manual. San Antonio, TX: Psychological Corporation.

[B48] Kwon S. Armstrong B. Wetoska N. Capan S. (2024). Screen time, sociodemographic factors, and psychological well-being among young children. JAMA Netw. Open 7:e2354488. 10.1001/jamanetworkopen.2023.54488 38441898 PMC10915694

[B49] Ladd G. W. Ettekal I. Kochenderfer-Ladd B. (2017). Peer victimization trajectories from kindergarten through high school: differential pathways for children's school engagement and achievement? J. Educ. Psychol. 109, 826–841. 10.1037/edu0000177

[B50] Larson K. Russ S. A. Nelson B. B. Olson L. M. Halfon N. (2015). Cognitive ability at kindergarten entry and socioeconomic status. Pediatrics 135, e440–e448. 10.1542/peds.2014-0434 25601983

[B51] Lawson G. M. Hook C. J. Farah M. J. (2018). A meta-analysis of the relationship between socioeconomic status and executive function performance among children. Dev. Sci. 21:e12529. 10.1111/desc.12529 28557154 PMC5821589

[B52] Lechner C. M. Bender J. Brandt N. D. Rammstedt B. (2021). Two forms of social inequality in students' socio-emotional skills: do the levels of big five personality traits and their associations with academic achievement depend on parental socioeconomic status?. Front. Psychol. 12:679438. 10.3389/fpsyg.2021.679438 34367000 PMC8335486

[B53] Leerkes E. M. Paradise M. J. O'Brien M. Calkins S. D. Lange G. (2008). Emotion and cognition processes in preschool children. Merrill Palmer Q. 54, 102–124. 10.1353/mpq.2008.000934409987

[B54] Letourneau N. L. Duffett-Leger L. Levac L. Watson B. Young-Morris C. (2013). Socioeconomic status and child development: a meta-analysis. J. Emot. Behav. Disord. 21, 211–224. 10.1177/1063426611421007

[B55] Linebarger D. L. Barr R. Lapierre M. A. Piotrowski J. T. (2014). Associations between parenting, media use, cumulative risk, and children's executive functioning. J. Dev. Behav. Pediatr. 35, 367–377. 10.1097/DBP.0000000000000069 25007059

[B56] Lipina S. J. Martelli M. I. Vuelta B. Colombo J. A. (2005). Performance on the A-not-B task of Argentinean infants from unsatisfied and satisfied basic needs homes. Rev. Int. Psicol. 39, 49–60.

[B57] Liu Q. Zhou N. Cao H. Hong X. (2020). Family socioeconomic status and Chinese young children'social competence: parenting processes as mediators and contextualizing factors as moderators. Child. Youth Serv. Rev. 118:105356. 10.1016/j.childyouth.2020.105356

[B58] Määttä S. Kaukonen R. Vepsäläinen H. Lehto E. Ylönen A. Ray C.. (2017). The mediating role of the home environment in relation to parental educational level and preschool children's screen time: a cross-sectional study. BMC Public Health 17:688. 10.1186/s12889-017-4694-9 28865436 PMC5581928

[B59] MacKinnon D. P. (2008). Introduction to Statistical Mediation Analysis. London: Routledge.

[B60] Martin K. J. Beck A. F. Xu Y. Szumlas G. A. Hutton J. S. Crosh C. C.. (2022). Shared reading and risk of social-emotional problems. Pediatrics 149:e2020034876. 10.1542/peds.2020-034876 34889450

[B61] Massaroni V. Delle Donne V. Marra C. Arcangeli V. Chieffo D. P. R. (2024). The relationship between language and technology: how screen time affects language development in early life—A systematic review. Brain Sci. 14:27. 10.3390/brainsci14010027 38248242 PMC10813394

[B62] Maxwell S. E. Cole D. A. (2007). Bias in cross-sectional analyses of longitudinal mediation. Psychol. Methods 12:23. 10.1037/1082-989X.12.1.23 17402810

[B63] McDonald K. L. Asher S. R. (2018). “Peer acceptance, peer rejection, and popularity: Social-cognitive and behavioral perspectives,” in Handbook of Peer Interactions, Relationships, and Groups, eds. Bukowski W. M. Laursen B. Rubin K. H., 2nd Edn. (New York: The Guilford Press), 429–446.

[B64] McLoyd V. C. (1998). Socioeconomic disadvantage and child development. Am. Psychol. 53, 185–204. 10.1037/0003-066X.53.2.185 9491747

[B65] Merz E. C. Zucker T. A. Landry S. H. Williams J. M. Assel M. Taylor H. B.. (2015). Parenting predictors of cognitive skills and emotion knowledge in socioeconomically disadvantaged preschoolers. J. Exp. Child Psychol. 132, 14–31. 10.1016/j.jecp.2014.11.010 25576967 PMC4355039

[B66] Miyake A. Friedman N. P. Emerson M. J. Witzki A. H. Howerter A. Wager T. D. (2000). The unity and diversity of executive functions and their contributions to complex “frontal lobe” tasks: a latent variable analysis. Cogn. Psychol. 41, 49–100. 10.1006/cogp.1999.0734 10945922

[B67] Moffitt T. E. Arseneault L. Belsky D. Dickson N. Hancox R. J. Harrington H.. (2011). A gradient of childhood self-control predicts health, wealth, and public safety. Proc. Natl. Acad. Sci. 108, 2693–2698. 10.1073/pnas.1010076108 21262822 PMC3041102

[B68] Noble C. H. Cameron-Faulkner T. Lieven E. (2018). Keeping it simple: the grammatical properties of shared book reading. J. Child Lang. 45, 753–766. 10.1017/S0305000917000447 29145915

[B69] Norfadillah W. Hutagalung F. Nor M. M. Isa Z. M. (2017). A study of socioeconomic status (SES) on cognitive abilities among preschoolers in Klang Valley, Malaysia. Adv. Sci. Lett. 23, 2141–2144. 10.1166/asl.2017.8579

[B70] Oshchepkova E. S. Shatskaya A. N. (2023). Developm ent of narratives in children aged 6–8 years depending on the level of executive functions. Lomonosov Psychol. J. 46, 11–284, 262. 10.11621/LPJ-23-36

[B71] Pfost M. Heyne N. (2023). Joint book reading, library visits and letter teaching in families: relations to parent education and children's reading behavior. Read Writ. 36, 2627–2264. 10.1007/s11145-022-10389-w

[B72] Pickering H. E. Peters J. L. Crewther S. G. (2023). A role for visual memory in vocabulary development: a systematic review and meta-analysis. Neuropsychol. Rev. 33, 803–833. 10.1007/s11065-022-09561-4 36136174 PMC10770228

[B73] Pons F. Harris P. (2000). Test of Emotion Comprehension: TEC. Oxford: University of Oxford.

[B74] Pons F. Harris P. L. De Rosnay M. (2004). Emotion comprehension between 3 and 11 years: developmental periods and hierarchical organization. Eur. J. Dev. Psychol. 1, 127–152. 10.1080/17405620344000022

[B75] Pons M. Bennasar-Veny M. Yañez A. M. (2020). Maternal education level and excessive recreational screen time in children: a mediation analysis. Int. J. Environ. Res. Public Health 17:8930. 10.3390/ijerph17238930 33271768 PMC7730269

[B76] Read K. Gaffney G. Chen A. Imran A. (2022). The impact of COVID-19 on families' home literacy practices with young children. Early Child. Educ. J. 50, 1429–1438. 10.1007/s10643-021-01270-6 34629842 PMC8488072

[B77] Rocha A. M. A. (2016). Compreensão e regulação das emoções: suas relações com a eficácia na interação social em criança. [Doctoral Dissertation]. Universidade de Lisboa, Portugal.

[B78] Roy M. G. Agrawal A. Patil R. Shrivastava J. (2024). Assessment of the risk of behavioral problems in children below five years in relation to screen time: a cross-sectional study. Cureus 16:e72459. 10.7759/cureus.72459 39600757 PMC11589389

[B79] Roy-Charland A. Lewis S. Pallister M. Richard J. Michaud M. Perron M. (2020). The use of dyadic reading in stimulating the comprehension of emotions. J. Genet. Psychol. 182, 75–88. 10.1080/00221325.2020.1868969 33413038

[B80] Salmon K. Reese E. (2016). Преимущества воспоминаний с маленькими. Curr. Dir. Psychol. Sci. 25, 233–238. 10.1177/0963721416655100

[B81] Schwering S. C. MacDonald M. C. (2020). Verbal working memory as emergent from language comprehension and production. Front. Hum. Neurosci. 14:68. 10.3389/fnhum.2020.00068 32226368 PMC7081770

[B82] Shatskaya A. Gavrilova M. Chichinina E. (2023). Voluntariness and type of digital device usage: a study in terms of Vygotsky's cultural–historical perspective. Front. Psychol. 14:1111613. 10.3389/fpsyg.2023.1111613 36949908 PMC10026563

[B83] Shehu B. P. (2019). Peer acceptance in early childhood: Links to socio-economic status and social competences. J. Soc. Stud. Educ. Res. 10, 176–200.

[B84] Sticca F. Brauchli V. Lannen P. (2025). Screen on= development off? A systematic scoping review and a developmental psychology perspective on the effects of screen time on early childhood development. Front. Dev. Psychol. 2:1439040. 10.3389/fdpys.2024.1439040

[B85] Uribe F. M. T. LeVine R. A. LeVine S. E. (2014). “Maternal behavior in a Mexican community: the changing environments of children,” in Cross-Cultural Roots of Minority Child Development (Psychology Press), 43–56.

[B86] van der Wilt F. (2024). The relation between social acceptance and school well-being in early childhood. Infant Child Dev. 33:e2470. 10.1002/icd.2470

[B87] Veraksa A. N. Almazova O. V. Bukhalenkova D. A. (2020). Executive functions assessment in senior preschool age: a battery of methods. Psikhol. Z. 41, 108–118. (in Russian). 10.31857/S020595920012593-8

[B88] Veraksa N. Veraksa A. Gavrilova M. Bukhalenkova D. Tarasova K. (2021b). The Russian version of the test of emotion comprehension: adaptation and validation for use in preschool children. Psychol. J. High. Sch. Econ. 18, 56–70. (in Russian). 10.17323/1813-8918-2021-1-56-70

[B89] Veraksa N. E. Veraksa A. N. Bukhalenkova D. A. Säljö R. (2021a). Exploring the development of executive functions in children in a digital world. Eur. J. Psychol. Educ. 37,1035–1050. 10.1007/s10212-021-00584-8

[B90] Veresov N. N. (2024). The history of development of the cultural-historical theory and its contemporary perceptions: answering questions and questioning answers. Lomonosov Psychol. J. 47, 223–255. 10.11621/LPJ-24-47

[B91] Vygotsky L. S. (1983). Collected Works, Vol. 3. Moscow: Pedagogika.

[B92] Vygotsky L. S. Cole M. (1978). Mind in Society: Development of Higher Psychological Processes. London: Harvard university press.

[B93] Wasik B. H. (1987). Sociometric measures and peer descriptors of kindergarten children: a study of reliability and validity. J. Clin. Child Psychol. 16, 218–224. 10.1207/s15374424jccp1603_626627889

[B94] Waters N. E. Ahmed S. F. Tang S. Morrison F. J. Davis-Kean P. E. (2021). Pathways from socioeconomic status to early academic achievement: the role of specific executive functions. Early Child. Res. Q. 54, 321–331. 10.1016/j.ecresq.2020.09.008 33519062 PMC7839968

[B95] Welsh M. C. Pennington B. F. Groisser D. B. (1991). A normative-developmental study of executive function: a window on prefrontal function in children. Dev. Neuropsychol. 7, 131–149. 10.1080/87565649109540483

[B96] Willoughby M. T. Kupersmidt J. B. Voegler-Lee M. E. (2012). Is preschool executive function causally related to academic achievement? Child Neuropsychol. 18, 79–91. 10.1080/09297049.2011.578572 21707258 PMC3417807

[B97] Wu Z. Hu B. Y. Wu H. Winsler A. Chen L. (2020). Family socioeconomic status and Chinese preschoolers' social skills: Examining underlying family processes. J. Fam. Psychol. 34:969. 10.1037/fam0000674 32406730

[B98] Xie Q. W. Chan C. H. Ji Q. Chan C. L. (2018). Psychosocial effects of parent-child book reading interventions: a meta-analysis. Pediatrics 141:e20172675. 10.1542/peds.2017-2675 29588337

[B99] Yang X. Chen Z. Wang Z. Zhu L. (2017). The relations between television exposure and executive function in Chinese preschoolers: the moderated role of parental mediation behaviors. Front. Psychol. 8:1833. 10.3389/fpsyg.2017.01833 29089912 PMC5651076

[B100] Zelazo P. D. (2006). The Dimensional Change Card Sort (DCCS): a method of assessing executive function in children. Nat. Protoc. 1, 297–301. 10.1038/nprot.2006.46 17406248

