# Supplementary material for: Bridging the gap: the impact of parental education and child leisure activities on cognitive and socioemotional skills in preschoolers
Source: Front Psychol. 2025 Aug 25;16:1568020. doi: 10.3389/fpsyg.2025.1568020 (PMC12414975; doi:10.3389/fpsyg.2025.1568020)
Supplement: Supplementary file 1 [file Table_1.DOCX]

**Supplementary Materials**

**Table 1**

Fit Indices for Each Mediation Model Test

| **Study variables** | **Direct Effects Model** | | | | **Partial Mediation** | | | | **Full Mediation Model** | | | |
| --- | --- | --- | --- | --- | --- | --- | --- | --- | --- | --- | --- | --- |
|  | RMSEA | CFI | TLI | SRMR | RMSEA | CFI | TLI | SRMR | RMSEA | CFI | TLI | SRMR |
|  | **Daily screen time as mediator** | | | | | | | | | | | |
| Visual working memory | 0 | 1 | 1 | 0 | 0 | 1 | 1 | 0 | 0.05 | 0.97 | 0.91 | 0.02 |
| Verbal working memory | 0 | 1 | 1 | 0 | 0 | 1 | 1 | 0 | 0.17 | 0.81 | 0.41 | 0.07 |
| Inhibition | 0 | 1 | 1 | 0 | 0 | 1 | 1 | 0 | 0 | 1 | 1 | 0.01 |
| Cognitive flexibility | 0 | 1 | 1 | 0 | 0 | 1 | 1 | 0 | 0.05 | 0.96 | 0.89 | 0.02 |
| Sociometric status | 0 | 1 | 1 | 0 | 0 | 1 | 1 | 0 | 0.04 | 0.97 | 0.92 | 0.021 |
| Emotion comprehension | 0 | 1 | 1 | 0 | 0 | 1 | 1 | 0 | 0.07 | 0.94 | 0.83 | 0.03 |
|  | **Shared book reading as mediator** | | | | | | | | | | | |
| Visual working memory | 0 | 1 | 1 | 0 | 0 | 1 | 1 | 0 | 0.05 | 0.94 | 1.08 | 0.02 |
| Verbal working memory | 0 | 1 | 1 | 0 | 0 | 1 | 1 | 0 | 0.20 | 0.54 | 0.38 | 0.08 |
| Inhibition | 0 | 1 | 1 | 0 | 0 | 1 | 1 | 0 | 0.01 | 0.99 | 0.99 | 0.01 |
| Cognitive flexibility | 0 | 1 | 1 | 0 | 0 | 1 | 1 | 0 | 0.04 | 0.94 | 0.81 | 0.02 |
| Sociometric status | 0 | 1 | 1 | 0 | 0 | 1 | 1 | 0 | 0.05 | 0.94 | 0.82 | 0.02 |
| Emotion comprehension | 0 | 1 | 1 | 0 | 0 | 1 | 1 | 0 | 0.09 | 0.8 | 0.39 | 0.04 |

**Table 2**

Сorrelations for children EF skills, emotion comprehension, sociometric status, screen time dimensions, frequency of shared book reading and level of parental education

| **Study variables** | **Values** | **1** | **2** | **3** | **4** | **5** | **6** | **7** | **8** | **9** |
| --- | --- | --- | --- | --- | --- | --- | --- | --- | --- | --- |
| Parental education (1) | Pearson's r | — |  |  |  |  |  |  |  |  |
|  | p-value | — |  |  |  |  |  |  |  |  |
|  | Spearman's rho | — |  |  |  |  |  |  |  |  |
|  | p-value | — |  |  |  |  |  |  |  |  |
|  | N | — |  |  |  |  |  |  |  |  |
| Shared book reading (2) | Pearson's r | **0.187***** | — |  |  |  |  |  |  |  |
|  | p-value | **<.001** | — |  |  |  |  |  |  |  |
|  | Spearman's rho | **0.199***** | — |  |  |  |  |  |  |  |
|  | p-value | **<.001** | — |  |  |  |  |  |  |  |
|  | N | **1288** | — |  |  |  |  |  |  |  |
| Daily screen time (3) | Pearson's r | **-0.271***** | -0.230*** | — |  |  |  |  |  |  |
|  | p-value | **<.001** | <.001 | — |  |  |  |  |  |  |
|  | Spearman's rho | **-0.273***** | -0.215*** | — |  |  |  |  |  |  |
|  | p-value | **<.001** | <.001 | — |  |  |  |  |  |  |
|  | N | **1280** | 1357 | — |  |  |  |  |  |  |
| Visual working memory (4) | Pearson's r | 0.05 | 0.006 | -0.021 | — |  |  |  |  |  |
|  | p-value | 0.081 | 0.847 | 0.46 | — |  |  |  |  |  |
|  | Spearman's rho | 0.039 | 0.004 | -0.008 | — |  |  |  |  |  |
|  | p-value | 0.175 | 0.888 | 0.79 | — |  |  |  |  |  |
|  | N | 1213 | 1218 | 1219 | — |  |  |  |  |  |
| Verbal working memory (5) | Pearson's r | **0.219***** | 0.121*** | -0.215*** | 0.315*** | — |  |  |  |  |
|  | p-value | **<.001** | <.001 | <.001 | <.001 | — |  |  |  |  |
|  | Spearman's rho | **0.187***** | 0.113*** | -0.227*** | 0.313*** | — |  |  |  |  |
|  | p-value | **<.001** | <.001 | <.001 | <.001 | — |  |  |  |  |
|  | N | **1246** | 1251 | 1251 | 2933 | — |  |  |  |  |
| Inhibition (6) | Pearson's r | 0.039 | 0.039 | -0.045 | 0.282*** | 0.223*** | — |  |  |  |
|  | p-value | 0.177 | 0.179 | 0.118 | <.001 | <.001 | — |  |  |  |
|  | Spearman's rho | 0.03 | 0.031 | -0.013 | 0.277*** | 0.209*** | — |  |  |  |
|  | p-value | 0.295 | 0.277 | 0.66 | <.001 | <.001 | — |  |  |  |
|  | N | 1202 | 1208 | 1210 | 2710 | 2737 | — |  |  |  |
| Cognitive flexibility (7) | Pearson's r | **0.069*** | 0.031 | 0.006 | 0.312*** | 0.293*** | 0.245*** | — |  |  |
|  | p-value | **0.014** | 0.264 | 0.823 | <.001 | <.001 | <.001 | — |  |  |
|  | Spearman's rho | **0.062*** | 0.027 | -0.001 | 0.322*** | 0.316*** | 0.244*** | — |  |  |
|  | p-value | **0.027** | 0.333 | 0.968 | <.001 | <.001 | <.001 | — |  |  |
|  | N | **1273** | 1283 | 1285 | 2950 | 2980 | 2832 | — |  |  |
| Sociometric status (8) | Pearson's r | 0.054 | -0.021 | 0.002 | 0.116*** | 0.113*** | 0.090*** | 0.091*** | — |  |
|  | p-value | 0.066 | 0.469 | 0.936 | <.001 | <.001 | <.001 | <.001 | — |  |
|  | Spearman's rho | 0.038 | -0.02 | -0.011 | 0.113*** | 0.117*** | 0.085*** | 0.102*** | — |  |
|  | p-value | 0.198 | 0.492 | 0.708 | <.001 | <.001 | <.001 | <.001 | — |  |
|  | N | 1150 | 1158 | 1161 | 2294 | 2330 | 2275 | 2419 | — |  |
| Emotion comprehension (9) | Pearson's r | **0.083**** | 0.013 | -0.081** | 0.204*** | 0.187*** | 0.122*** | 0.188*** | 0.037 | — |
|  | p-value | **0.004** | 0.665 | 0.005 | <.001 | <.001 | <.001 | <.001 | 0.082 | — |
|  | Spearman's rho | **0.059*** | 0.006 | -0.070* | 0.196*** | 0.185*** | 0.107*** | 0.204*** | 0.036 | — |
|  | p-value | **0.041** | 0.831 | 0.016 | <.001 | <.001 | <.001 | <.001 | 0.09 | — |
|  | N | **1185** | 1188 | 1189 | 2773 | 2816 | 2744 | 2823 | 2222 | — |
